# Non-physician clinicians in rural Africa: lessons from the Medical Licentiate programme in Zambia

**DOI:** 10.1186/s12960-017-0233-0

**Published:** 2017-08-22

**Authors:** Jakub Gajewski, Carol Mweemba, Mweene Cheelo, Tracey McCauley, John Kachimba, Eric Borgstein, Leon Bijlmakers, Ruairi Brugha

**Affiliations:** 1 Royal College of Surgeons in Ireland, 123 St Stephens Green, Dublin 2, Ireland; 2 Surgical Society of Zambia, Department of Surgery, University Teaching Hospital, Nationalist Road, Lusaka, Zambia; 3 College of Medicine, Malawi, Mahatma Gandhi, Blantyre, Malawi; 4 Radboud University Medical Centre Netherlands, Geert Grooteplein Zuid 10, 6525 Nijmegen, GA Netherlands

**Keywords:** Surgery, Non-physician clinicians, Surgical training, Health workforce retention, Medical licentiates

## Abstract

**Background:**

Most sub-Saharan African countries struggle to make safe surgery accessible to rural populations due to a shortage of qualified surgeons and the unlikelihood of retaining them in district hospitals. In 2002, Zambia introduced a new cadre of non-physician clinicians (NPCs), medical licentiates (MLs), trained initially to the level of a higher diploma and from 2013 up to a BSc degree. MLs have advanced clinical skills, including training in elective and emergency surgery, designed as a sustainable response to the surgical needs of rural populations.

**Methods:**

This qualitative study aimed to describe the role, contributions and challenges surgically active MLs have experienced. Based on 43 interviewees, it includes the perspective of MLs, their district hospital colleagues—medical officers (MOs), nurses and managers; and surgeon-supervisors and national stakeholders.

**Results:**

In Zambia, MLs play a crucial role in delivering surgical services at the district level, providing emergency surgery and often increasing the range of elective surgical cases that would otherwise not be available for rural dwellers. They work hand in hand with MOs, often giving them informal surgical training and reducing the need for hospitals to refer surgical cases. However, MLs often face professional recognition problems and tensions around relationships with MOs that impact their ability to utilise their surgical skills.

**Conclusions:**

The paper provides new evidence concerning the benefits of ‘task shifting’ and identifies challenges that need to be addressed if MLs are to be a sustainable response to the surgical needs of rural populations in Zambia. Policy lessons for other countries in the region that also use NPCs to deliver essential surgery include the need for career paths and opportunities, professional recognition, and suitable employment options for this important cadre of healthcare professionals.

## Background

Surgery has for long been neglected in sub-Saharan Africa (SSA) [1], where ratios of less than 1 surgeon per 100 000 population in most countries mean that around 95% of the population has no access to essential surgical services [2]. Elective and emergency general surgery is usually only available in urban hospitals staffed with specialist surgeons [3]; and lack of essential support staff and functioning equipment are additional reasons for limited access to safe surgery at district hospitals [4, 5]. To address the rural health workforce gap, the World Health Organization has recommended ‘task shifting’ [6], which is a solution used in high-income countries since the nineteenth century [7, 8]. Task shifting in delivering HIV/AIDS care reportedly results in good or comparable patient outcomes [9]. However, surgical task shifting, the ‘shifting of tasks from surgeons and anaesthesiologists to non-specialists’ [10], has raised ethical concerns about quality of care and patient safety [11, 12], although resistance has also been attributed to professional self-interest and competition [13, 14]. Despite these concerns, and because of the lack of alternatives [15], as well as evidence of economic benefits [16], task shifting of the delivery of emergency obstetrical interventions (including caesarean sections) and general surgery to non-physician clinicians (NPCs), working at district hospitals in underserved rural areas, has expanded [17, 18].

Zambia, with an estimated population of 16.2 million with 60.5% living in rural areas [19], faces health workforce shortages [20] and uses NPCs as the main surgical workforce at the district level [21]. In 2002, a 2-year Advanced Diploma Medical Licentiate (ML) programme was started for NPCs who had a minimum of 2 years’ experience following their basic 3-year NPC training [22]. The 2-year ML curriculum includes Biomedical Sciences, followed by hospital rotations for supervised training in Internal Medicine, Surgery, Paediatrics and Obstetrics and Gynaecology, followed by a 1-year internship. The aim was to produce a mid-level cadre to manage general and emergency cases in these four specialties, filling a critical gap due to shortages and absence of medical officers (MOs) [22]. Over 200 MLs were trained and in 2013 the programme was upgraded to the level of Bachelor of Science (4 years direct entry programme).

Studies elsewhere of NPC cadres to whom surgical care was ‘task-shifted’ suggest real benefits in terms of better access to safe surgery for rural populations, though primarily in obstetrics. In Mozambique, almost all caesarean sections, emergency hysterectomies and laparotomies for ectopic pregnancy outside of the main urban areas are done by NPCs, called ‘técnicos de cirurgia’ [16], with a similar practice in Malawi [23]. Despite reports of their positive impact on population access to surgery, NPCs face significant challenges [24], including lack of recognition by medical doctors, resulting in marginalisation and loss of motivation [25]. Lack of career opportunities [26] and low salary packages are perceived by them as unfair [27]. A study from Tanzania reported that low motivation among NPCs, stemming from an experience of low social status and remuneration, was associated with the provision of poor quality of care [28].

The Zambia Human Resources for Health Strategic Plan (2006–2010) aimed for MLs to have a defined career structure that would retain them within the health system and improve primary health care delivery [29]. This paper examines the roles and experiences of MLs delivering surgical care at district-level hospitals in Zambia. To date, the achievements and challenges of this programme have not been evaluated, and this study aims to fill this knowledge gap. The paper examines the roles and experiences of MLs delivering surgical care at district-level hospitals in Zambia, also including the perspectives of their colleagues, supervisors and trainers.

The study was conducted as part of the Clinical Officers Surgical Training in Africa (COST-Africa) project that aimed to strengthen surgical systems at the district level through the training, deployment and supervision of NPCs in Malawi and Zambia. The research objectives were to measure the impact and cost-effectiveness and explore the feasibility, sustainability and possible benefits of surgical ‘task shifting’ to NPCs. In Malawi the project supported the establishment of a Bachelor of Science in general surgery at the University of Malawi’s College of Medicine; and sponsored the first cohort of 16 NPC surgical trainees who received in-service training at eight district hospitals, delivered by visiting surgical specialists. In Zambia, COST-Africa, working in collaboration with Chainama College of Health Sciences (CCHS), designed a 3-month intensive training programme, mainly focused on essential surgical skills for already graduated MLs. Upon completion of the training, 10 MLs were deployed to nine district-level hospitals across Zambia where no ML had previously been posted. Provincial specialist surgeons were assigned to make periodic supervisory visits to these hospitals to supervise and mentor the MLs. Impact of their presence was measured over the period of 2 years (quantitative paper forthcoming). This study was conducted as part of the evaluation of COST-Africa, looking broadly at issues related to the ML profession in Zambia. It was guided by two main questions: (1) what is the contribution of MLs to surgery at district-level hospitals? and (2) what are the experiences and challenges faced by MLs in these settings?

## Methods

A qualitative approach, utilising in-depth interviews, explored the challenges experienced by MLs (mostly participants of the COST-Africa project) working at the district level; their perceptions of their career prospects; and also the perspectives of their hospital colleagues, surgeon supervisors and national training bodies on the contribution of surgically trained MLs. A generic topic guide for all groups of participants was developed, based on interactions of COST-Africa researchers with the MLs, their supervisors and a literature review. The topic guide was piloted with MLs.

### Sampling

National-level interviewees were members of the task force constituted to plan implementation of the ML programme in the early 2000s. Interviewed surgeons were members of a surgical oversight team who visited COST-Africa trained MLs in their hospitals for training and supervision purposes. During the visits, the surgeons interacted with hospital managers and other staff and gained insight into how the presence of MLs impacted on surgery at the hospitals. Interviewed MLs included COST-Africa trainees and other MLs working at hospitals included in the study. The trainees were part of the final cohorts of NPCs to obtain the ML Diploma prior to the introduction of a new BSc programme in 2013. No incentive was provided to study participants. Table 1 tabulates the levels, categories and numbers of interviewees (*n* = 43).

**Table 1:** Number of research participants

Level	Function	Number of participants
National	Training bodies representatives (TR)	5
Provincial	Surgeons (SU)	5
Level 1 (district and mission hospitals)	Medical licentiates (ML)	12
	Human resources officers (HRO)/hospital administrators (HA)	8
	Theatre nurses (NU)	2

### Data collection

All interviews were conducted by the first and second author (JG, CM) near the end of the project, in the second half of 2015, during hospital visits; at the last of the 6-month meetings with the surgical oversight team; and at the offices of the relevant national bodies. Before each interview, which typically lasted about 1 h, participants were given information about the purpose of the interview and types of questions that would be asked. The consent process gave interviewees the assurance of full anonymity for all information provided.

### Data analysis

All interviews were audio-recorded and transcribed using MS Word and analysed in NVivo v.11 [30]. Two project-employed researchers jointly designed a data coding frame, using the ‘top-down’ method of theme identification [30, 31]. In addition, the literature review and prior experience in interacting with the MLs and hospital staff guided the researchers towards the most relevant areas, without the need to ‘discover’ the codes de novo. As a first step, the researchers coded the data based on the pre-developed coding scheme. In the second step, additional codes were developed that were considered relevant to the research questions. Then the codes were discussed and agreed among the wider team of researchers to build consensus on the most relevant themes. In the last step the final structure of themes and subthemes was established.

## Results

Findings are presented in two sections representing sets of themes that describe the realities of MLs’ work at the district level, which the two main research questions sought to answer. Findings’ subsections reflect these themes. Quotations are selected to highlight the summary points presented in text above each of them. They are anonymous with profession and an identification number given in brackets.

### ML contribution at district-level hospitals

#### Benefits of surgical task shifting

All participants, especially hospital managers and MOs, reported that MLs were highly valued and essential to the delivery of surgery at the district level. MLs took much of the burden of work from other cadres, particularly MOs, because they were clinically trained and surgical and medical duties could therefore be delegated to them:

I can tell you there was a huge difference. It was really helpful and I had time to rest sometimes. (MO 11)There is also an improvement on the workload for doctors because he is in charge of the maternity ward. (HA 1)

Surgical training and skills distinguished MLs from NPC cadres with less-advanced clinical skills. The deployment of MLs to work in the operating theatre (OT) departments of district hospitals freed up MOs to undertake other clinical and administrative duties. Only one MO felt that an ML colleague was neglecting medical duties in favour of surgery, whereas all other MOs viewed MLs as having a positive impact on the availability of surgery. Levels of ML absenteeism from district hospitals were reportedly lower than for MOs, who were frequently away attending workshops or meetings, which was the main reason why MOs spent less time in OT than did MLs.

Medical doctors tend to be in meetings most of the time, but a medical licentiate can be handling those cases (...) they rarely spend time in meetings. (HRO 3)

Representatives of the national training bodies emphasised that the type of training—specialising in clinical practice at district level—received by MLs reduces the likelihood of their being diverted from clinical practice, which often happens to MOs.

Doctors, as we are producing them, are not keen on clinical practice. That is why we have hundreds of them going into public health. Now who remains in the hospital? MLs, because they are crafted for practice. (TR 2)

In most hospitals, the arrival of a surgically trained ML was reported to have facilitated increases in the volume of simple surgical procedures. MOs, who worked alongside MLs, gave examples of surgical procedures where MLs made an important contribution after taking up posts at district-level hospitals. These included life-saving surgery, such as emergency hysterectomies, and elective surgery procedures not previously delivered at these hospitals.

This year, 200+ cases have been done which is commendable with the coming of the ML in comparison with what was done the previous year – which was about 110 for the whole year. (MO 10)Especially the laparotomies, intestinal obstruction or anything else to do with the abdomen, we would do them here when he was around (MO 8)

Reductions in unnecessary referrals were reported in all of the hospitals, which were attributed to the arrival of the surgically trained ML. These included relatively simple emergency cases such as appendicitis and strangulated hernias that surgically trained MLs can undertake. This was seen as an important cost saving.

We used to refer hernias, but now we are able to do hernias and fibroids at the hospital. This means that the hospital has reduced fuel expenses that we previously paid as a result of high number referrals. (MO 9)A lot of procedures including those they (surgical staff) never used to handle are now being handled. (HA 5)

#### Surgical skills and leadership

Colleagues reported that trained MLs had practical skills at a level comparable to MOs that allowed them to undertake most of the clinical duties required at district-level hospitals. The extent of training these two cadres received was the only major difference noted by the interviewees; and some of the MOs stated that the MLs were more skilled in surgery than they were themselves. The MLs’ surgical expertise was attributed to (a) the exposure they got from working in rural hospitals, (b) experience from working in higher-level facilities where they were trained by qualified surgeons (part of the mandatory ML training rotations) and (c) the rigorous practice-based ML training with a focus on surgical skills, which the 3-month additional COST-Africa training had reinforced.

They also are able to do surgery such as caesareans, ovarian cysts, hernias, even other things not done by doctors, but that MLs are able to do because of their rigorous training – some have had the opportunity to pass through the hands of very senior surgeons. (MO 10)

Some study participants reported that their surgical skills placed MLs in unexpected positions of leadership of the hospital surgical department, despite being junior to MOs who often did not have the same level of expertise or experience. In some hospitals, MLs undertook the cases that needed more sophisticated surgical skills, leaving MOs to do simpler cases.

Ideally, the medical doctor is supposed to do more of the bigger things and the ML should do the smaller things. But the way it used to be, the ML would do most of the bigger things especially in theatre. (MO 8)

#### Capacity building at the district level

MLs also played an important role in informal on-the-job surgical training of medical officers and other clinicians such as clinical officers at district-level hospitals. This included assisting newly trained and newly deployed MOs in gaining experience in surgery; and imparting new surgical skills to, and enhancing the scope of surgical practice of, well-established district-level MOs.

Even me when I came here, I wasn’t so conversant with the caesarean sections and other small procedures, but I would always ask that he [ML] shows me how to carry out certain procedures. (MO 8).

The process of passing on surgical skills from MLs to other cadres was reported in most of the hospitals. However, in some places, this cascade effect did not happen because some MOs were reluctant to seek advice from more surgically experienced MLs who were junior to them.

#### Retention in rural areas

Findings suggest that MLs were more likely than MOs to remain working in district and other rural hospitals, because their professional training was tailored to fit the district needs. Only one ML expressed an intention to relocate soon so as to seek employment in urban areas. The others reported that if they were posted to higher-level (referral) hospitals, they would be ‘overshadowed’ by specialists, surgical trainees and newly qualified MOs, seeking to upskill surgically; and thereby they would lose their access to the operating theatre and opportunities for using their surgical skills.

The ML programme was designed to serve the district communities and we have an understanding of that, so leaving the district for me is not an option unless once I specialize in something and am given an opportunity to work in a particular field. (ML 8)

MLs were aware of their limitations in terms of surgical skills and other clinical competencies. However, at this point in Zambia, there was no possibility for them to specialise in any clinical discipline. This meant that their only opportunity to utilise and develop their clinical skills lay at district-level hospitals rather than at higher-level facilities.

I'm more useful at district hospitals as opposed to bigger hospitals where there are consultants and senior doctors, because you tend not to be of much use there. (ML 4)

### Challenges of MLs in district hospitals

#### Limited recognition of MLs’ competencies

Zambia’s Medical Licentiate programme, which started in 2002, is well established. However, study participants (national stakeholders, surgeons, as well as MLs) pointed out that in some hospitals the MLs lacked recognition from some MOs for their clinical and surgical skills. This limited their opportunities to carry out specific surgical procedures, which they had been trained to deliver.

Some MLs said that the Advanced Diploma qualification, which was lower than a BSc, contributed to their low status; it was not commensurate with their level of training and scope of practice. In some hospitals, MOs and qualified expatriate surgeons who were not conversant with the ML training, questioned their capacity to undertake particular surgical procedures. This lack of recognition translated into complaints that MLs brought to the attention of their supervising surgeons. An interviewed surgeon quoted an ML’s complaints as follows:

“I am having challenges because there are cases I know I can do, but because there is someone who is senior to me, they have been referring those cases”; So when an ML says: “I can do this”, someone would say “who does he think he is”. (SU 1)

Lack of knowledge of the role, training and individual competencies of MLs was not the only problem faced by them. Both MLs and doctors reported that some other doctors felt their professional position was threatened by MLs. Such doctors avoided consulting with MLs about cases where the ML might have more experience as well as the training to manage the case, sometimes resulting in referral of cases that could have been managed at the district hospital.

“...doctors fail to ask for advice because they feel somewhat belittled when they think that they have to ask for help from someone less qualified than they are”. (MO 6).

Low recognition of the ML profession was also attributed by some study participants to lack of representation at higher levels of the health system administration, both at the provincial and national directorate levels in the Ministry of Health, which made it difficult for MLs to air and resolve systemic grievances:

Because of similarities in things that we do with medical doctors, our representation [at ministerial level] has been lumped under clinical care, which is unfortunate. (...) Then at provincial level, there must also be a representative who understands issues that pertain directly to MLs. (TR 3)

MLs felt that they were left behind in national planning, and at the regional and district levels, there was no dedicated office they could go to raise issues or seek assistance.

#### Lack of career progression of MLs

Lack of a clear career path was seen by some study participants as an important reason for some MLs leaving the profession. This stemmed from the design of the ML programme in the early 2000s, where no clear career path or opportunities for progression in clinical practice were agreed. The only available option to progress was to embark on a medical degree, which was an unattractive option for most MLs because the University of Zambia did not offer any exemptions based on experience or years of practice as a ML. Consequently, some MLs had opted to leave the public sector to enter private practice, opt for more financially attractive career paths outside of clinical practice, or leave the ML profession.

It was a natural progression. People have to progress in their careers all the time especially looking at the number of years and the responsibilities that one has as an ML. (TR 3)

Despite having training and experience in surgery and being valuable for communities in rural areas of Zambia, some MLs pursued new educational programmes for the sake of having a degree qualification, because opportunities for advancement were clearer to them in such fields.

We realized that most of them moved out from the ML profession or the clinical officer profession to social sciences such as Development Studies, Public Administration or Human Resources, because that was the only way they could get degree qualifications. (TR 1)

While the new BSc in Clinical Sciences was being established at the time of these interviews, there were mixed feelings about the BSc among MLs who participated in our study; they were uncertain as to what it would mean for them. They reported that the curriculum covered similar areas to their original ML training; and that it would not provide them with new surgical techniques or knowledge beyond what they had learned at their district hospitals.

To be frank I haven’t seen much new things, regarding this program. The only thing I can say is it has just given me an opportunity to work with more qualified people like consultants, but otherwise there is nothing really different from the programme that we did. (ML 4)

This illustrates a feeling among MLs that their original training already gave them competencies at the BSc level; and it was not yet clear if the degree would offer a career path beyond that available to ML diploma-holders. Their concerns pertained to lack of clear promotion guidelines or higher salary levels for MLs holding a BSc. In Zambia there was a limited number of government-approved and government-funded posts. Therefore, a degree (or diploma) holder was not automatically put on a salary scale reflecting his or her qualifications.

As you know our major employer is the government. So far, there is no establishment for the BSc because it hasn’t yet been absorbed into the system; probably after producing the first intake, the salaries will change or not. Even as an ML, it was not automatic for me to be put on the payroll, so we don’t know what will happen for this programme. There will be one adjustment on the paper but the opportunities it will bring nobody knows. (ML 10)So when one graduates from the BSc programme, there is no progression; no career progression. (TR 3)

On the other hand, some MLs were optimistic about the BSc as they saw it as a way of becoming more appreciated as individuals in district hospitals, because they were already expected to do major surgery beyond what a diploma holder should do; and in their view, the BSc degree would reflect their competencies and improve their status at work.

I think with the BSc programme, there will be some respect, with what you offer the community as well as what you are earning. I will also be considered as a senior officer at the hospital level. The training level will also enhance my skills, meaning I will be valuable at the district level. (ML 8)

## Discussion

This paper adds to the literature on task shifting of surgery to NPCs in sub-Saharan Africa, from Mozambique [27] and Malawi [32], helping to identify a sustainable response to the surgical needs of rural populations. It provides new evidence that helps define the role and benefits of the NPC model, through the perspective of those who work most closely with the MLs. The uniform view was that MLs were providing an essential service that would otherwise be inaccessible for most rural populations. Supervising surgeons stated that MLs’ surgical competencies and practice were appropriate to district-level hospitals. Most medical colleagues (general MOs) attested to their surgical skills, which they saw as often superior to that of most MOs working at district-level hospitals. Hospital managers valued the costs saving from fewer referrals of simple cases that could be done locally, confirming studies from Mozambique [27], Niger [33], Senegal [34] and Malawi [35]. Their supervising surgeons valued the benefits of reductions in unnecessary referrals, freeing up specialist surgeons in provincial hospitals to deliver specialist surgery.

The study, through an in-depth exploration of the impact of MLs at district-level hospitals, identified additional benefits, beyond the essential emergency and elective surgical services provided by MLs to rural dwellers. It has been hypothesised that NPCs in Africa provide surgical training to their physician colleagues [36]. Our study provides data supporting this hypothesis. MOs as well as MLs reported that MLs strengthened existing and imparted new surgical skills to other hospital clinicians, including MOs who had higher-level qualifications. This ‘on-the-job’ training of MOs is beneficial to them in the absence of continuing professional development or supervised mentoring of district-level surgically active clinicians, which is rarely if ever available in these settings [37]. This benefit could be attributed to the practical training undergone by MLs, who spent 16 months of their advanced diploma training in (4 × 4 month) attachments in the four primary clinical specialties—medicine, surgery, obstetrics/gynaecology and paediatrics. These attachments were based at busy provincial and large mission hospitals, under the direct supervision of qualified specialists, followed by a 1-year internship at district-level hospitals. This practical training complemented and contrasted with that of MOs, who lacked the same level of district and rural clinical exposure.

The presence of surgically trained and experienced MLs at district-level hospitals had two additional, indirect benefits: the first was task-sharing [38], in that MLs reduced the work burden, including routine surgery that would otherwise fall on MOs. One of the consequences of excessive workload is ‘brain drain’, which disproportionately impacts on health workers in rural areas [39]. A second indirect benefit, which has been reported by others [40, 41] and which our findings support, is that the lack of a medical qualification makes for a more stable clinician-cadre that is more likely to be prepared to live in rural areas and remain working at district-level hospitals. Based on the views of national and district respondents, Zambia’s ML programme is achieving its aim of filling the health workforce gap in rural areas [42], where qualified surgeons and experienced MOs are unlikely to remain, because of the more attractive career options available to them in urban Zambia and abroad.

Zambia’s ML programme, as it was in 2015, is still far from a panacea in terms of meeting the essential surgery needs of district and rural populations. While a BSc programme had been introduced in the 2 years prior to the interviews, MLs still lacked a clear career path; and it was not yet evident if the BSc would be sufficient to stem an outflow of NPCs out of the public sector into other professions and jobs with better career progression opportunities. Similar problems of unclear career paths for NPCs have been reported in other countries [32, 43]. While the recently introduced BSc might result in a higher pay grade, at least for some, there was some scepticism in the ML community in Zambia about their longer-term prospects, based on past experience. Reported complaints in this study that the content of the BSc curriculum went little beyond its Higher Diploma predecessor may be limited to this specific group of ML interviewees of whom some were the pioneers in a transition from the Diploma to a BSc degree.

Resistance of MOs towards allowing MLs to practice major surgery can have its roots in fears about possible competition, loss of prestige and financial benefits; and threats to the autonomy of the medical profession [18], as much as concerns about patients’ safety [14]. In this study, the non-MLs who were interviewed—MO colleagues, hospital human resource managers and administrators, and supervising surgeons—had direct knowledge of the performance, strengths and weaknesses of the surgically active MLs; and none expressed resistance to their role as surgeons. However, these positive relationships between MOs and MLs may not be fully representative of district hospitals, nationally, in that regular supervisory visits by specialist surgeons are likely to have had a broader positive impact on the district hospital.

Despite these largely positive perceptions, MLs were not perceived as partners by some MOs. Also, MLs in some hospitals felt restrained from carrying out surgery for which they had been trained, because of their lower qualification and MOs’ limited understanding of their capabilities. Unresolved issues around seniority, role definition and the scope of decision-making capacity and practice boundaries have also been reported in other studies [27]. MLs working at district-level hospitals face professional recognition challenges that could be avoided through sensitisation of MOs and hospital managers, making them aware of MLs’ competencies (and their limitations); ensuring that they get assigned to clinical duties as befits their skill-sets; and by putting in place effective district hospital surgical team supervision and support. Training of other cadres should also incorporate knowledge about newly established professions such as MLs, where they work alongside each other.

### Study limitations

This study provides new knowledge and supports published evidence about the role of NPCs in the delivery of surgical services in rural sub-Saharan Africa. This evidence, from one country context (Zambia), has several limitations. Firstly, although this paper provides data about task shifting and describes its benefits, supporting the case for using NPCs in situations where trained surgeons are not available, it does not aim to report on the surgical competencies of MLs at district-level hospitals. A separate paper will report and evaluate the supervisory processes and quality assurance systems that the project implemented.

Second, 10 of 12 MLs interviewed in this study participated in the COST-Africa project, which provided them with an additional 12-week course in surgical skills; and they benefited from three to six monthly supervised visits for surgical specialists during their deployment to nine district-level hospitals, where the interviews of surgeons and hospital managers all took place. This limits the representativeness of the sample and the findings, because this group of MLs was expected to have more surgical training than those who only underwent the basic ML training. The presence of supervising surgeons could have also helped to establish good relationships between MLs and MOs in supervised hospitals. However, the surgical skills of other MLs practicing in Zambia were unlikely to be significantly different from those of the project’s trainees, and interviews incorporate the participants’ views of MLs more broadly, and not only of those who were part of the COST-Africa project.

Third, the project did not (seek to) ascertain the views of those national stakeholders who were not directly familiar with the ML programme—all those interviewed were involved in and hence had direct knowledge of it. This was consistent with the aim of the study, which was to deepen understanding about the role and potential of surgically active MLs at district-level hospitals in Zambia, drawing on first-hand and observed experience. The more negative views of surgical task shifting to NPCs on the part of others—doctors who were less familiar with the programme—may point to a selection bias in those interviewed. However, the views of those who directly worked with and supervised the MLs can be considered a more valid perspective.

Fourthly, we did not triangulate data collection methods in our study, using interviews as the only method to capture data; however, using interviews to capture a wide range of perspectives was appropriate to the research questions in this paper. Finally, 10 of 12 of the interviewed MLs were a transitional group, some of whom were transitioning or bridging from the earlier ML programme to the new BSc programme, which gave them a particular perspective on career paths for MLs. A study conducted at a later point with NPCs who had undertaken a BSc as their primary qualification might have produced different perspectives.

## Conclusions

In the light of the persistent, and in some countries the difficult to resolve, shortages of qualified surgeons in district and rural parts of many sub-Saharan African countries, the deployment and supervision of surgically trained NPCs to district hospitals is one strategy for making surgery available in rural areas. This is an option that has, hitherto, been contentious [14], with limited evidence of its safety and effectiveness. This article provides evidence that the introduction of advanced clinical (including surgical) training, on top of the basic 3-year NPC training programme is acceptable to and supported by the principal district stakeholders and can increase the volume and scope of surgery delivered. It also supports the view that NPC training programmes, and perhaps the evolution from a Diploma to a BSc programme, could contribute to the retention of a ‘fit-for-purpose’ clinician cadre that meets the healthcare needs of rural populations in Africa.

The sustainability of the ML profession in Zambia is not assured; and more work needs to be done to ensure appropriate professional recognition; and longer-term career paths for those with the ability and ambition to pursue them. There is also a need to embed the ML profession within the district hospital system, giving more attention to defining appropriate roles for MLs with BScs. Surgical team-leadership and district hospital team-building, requires better definition of the roles and responsibilities of the different clinical, nursing, management and support cadres, with attention to their career needs and paths. Job descriptions, founded on a better understanding of their strengths, limitations and potential, are particularly important, because it would give them assurance about their competences and protect them in case of legal challenges related to the scope of their practice. The above recommendations may be useful to policy makers, if sustainable solutions to the health care needs of Africa’s district and rural populations are to be put in place.

## Abbreviations

BSc: Bachelor of Science
CCHS: Chainama College of Health Sciences
COST-Africa: Clinical Officer Surgical Training in Africa
HA: Hospital administrator
HRO: Human resources officer
ML: Medical licentiate
MO: Medical officer
NPC: Non-physician clinician
NU: Theatre nurse
OT: Operating theatre
SSA: Sub-Saharan Africa
SU: Surgeon
TR: Training body representative
